# The two-tales of smoking: aberrations in sperm parameters and failure in assisted reproduction

**DOI:** 10.1080/2090598X.2022.2090135

**Published:** 2022-06-23

**Authors:** M. A. Arshad, A. Zil-E-Ali, M. T. Iqbal, A. Majzoub

**Affiliations:** a The Children’s Hospital and Institute of Child Health, Multan, Pakistan; b Pennsylvania State University College of Medicine, Hershey, PA, USA; c Fatima Memorial Hospital, Lahore, Pakistan; d Department of Urology, Hamad Medical Corporation, Doha, Qatar; e Weill Cornell Medicine- Qatar, Doha, Qatar

According to the American Society for Reproductive Medicine, failure to achieve pregnancy after regular and unprotected sexual intercourse for 12 months is defined as infertility. It is known that 15% of all couples attempting natural conception face difficulty and male infertility is believed to contribute to almost 50% of cases [1]. Evidence suggests that among male-related risk factors leading to infertility, smoking plays a significant contribution. With the exception of few controversial studies, the negative impact of smoking on semen quality including sperm count, motility, and morphology is well documented [2–4]. In a meta-analysis done by Bundhun et al., the authors revealed that oligospermia (relative risk: 1.29, P = 0.02) and morphological defects (mean difference [MD]: 2.44, P = 0.001) were significantly higher among smokers compared with non-smokers [5]. Another meta-analysis by Sharma et al. including 5865 participants similarly reported significant reduction in sperm count (MD: −9.72 × 106/ml), motility (MD: −3.48%), and morphology (MD: −1.37%) among participants exposed to cigarette smoking versus non-smokers. The authors further revealed that the effect size was higher in infertile men and in those with moderate/heavy exposure than the general population [6].

Among the various mechanisms linking smoking with altered semen parameters, seminal oxidative stress is most commonly investigated. This imbalance in redox potential is the result of aggravated production of reactive oxygen species (ROS) coupled with minimal antioxidant repairing mechanisms in the spermatozoa [7]. Oxidative stress can impair sperm quality as it can incite lipid peroxidation, aggravate abortive apoptosis and result in high sperm DNA fragmentation. The degree of oxidative stress is directly related to higher rates and duration of cigarettes consumed by the smoker [2,8].

The alteration in essential minerals such as zinc is another mechanism with which smoking can impair semen quality. Zinc is vital for the process of spermatogenesis and its deficiency may halt the process and additionally impact serum testosterone production [8,9]. Liu et al. reported lower levels of zinc in the semen of smokers who also had significantly lower sperm parameters compared with non-smokers [10]. Another study by Bazid et al. identified significant negative correlation between seminal zinc levels and smoking index and a significant positive correlation between zinc levels and sperm motility and viability [11].

Some authors have advocated that the harmful effects of cigarette smoking may be attributed to nicotine rather than the toxic compounds contained within it. Animal and human studies have reported significantly negative effects for nicotine and its metabolites (cotinine/trans-3’-hydroxycotinine) on semen parameters, particularly motility and viability [12,13]. These findings indicate that nicotine in inhalational or oral form could affect fertility. However, these changes were not permanent and improved 30 days after cessation of oral nicotine, suggesting an element of reversibility [12].

Nowadays, assisted reproduction has offered infertile couples a decent chance of conception. Nonetheless, recent evidence indicates that the detrimental impact of smoking on sperm function can influence the outcome of assisted reproduction. One step further in investigating the effects of smoking on sperm physiology, Kovac et al. revealed that smoking could also influence the outcomes of in-vitro fertilization (IVF) and intracytoplasmic sperm injection (ICSI). They reported significantly lower live birth rates in couples whose male partners recently smoked (7.8%) compared with those non-smoking couples (21.1%) [1]. Another study by Joesbury et al. investigated the impact of smoking among both couples on assisted reproductive outcomes. The authors particularly found a significant relationship between male age and smoking and embryo quality. Their results revealed that with every single year of increasing male age, a 2.4% reduction in the possibility of pregnancy can be expected [14].

The current literature confidently demonstrates the detrimental impact of smoking not only on natural fertility potential but also on the outcomes of assisted reproduction [5]. These findings extend the health benefits of smoking cessation from prevention of cardiopulmonary and/or systemic pathologies to enhancement of reproductive health. Successful interventions aiming to help patients refrain from smoking should be implemented and further research is required to understand the psychosocial reasons for nicotine consumption, and to explore different interventions that could help patients quit smoking.
